# Supplementary material for: Vast heterogeneity in cytoplasmic diffusion rates revealed by nanorheology and Doppelgänger simulations
Source: Biophys J. 2023 Feb 3;122(5):767–83. doi: 10.1016/j.bpj.2023.01.040 (PMC10027447; doi:10.1016/j.bpj.2023.01.040)
Supplement: Document S1. Figures S1–S6 [file mmc1.pdf]

Biophysical Journal, Volume *122*

Supplemental information

Vast heterogeneity in cytoplasmic diffusion rates revealed by nano-rheology and Doppelgänger simulations

Rikki M. Garner, Arthur T. Molines, Julie A. Theriot, and Fred Chang

Vast heterogeneity in cytoplasmic diffusion rates revealed by nanorheology and Doppelgänger simulations

Rikki M. Garner[1,2,4,*,&], Arthur T. Molines[3,4,*], Julie A. Theriot[1,2,4,#], and Fred Chang[3,4,#]

1. Biophysics Program, Stanford University School of Medicine, Stanford, CA, USA 2. Department of Biology and Howard Hughes Medical Institute, University of Washington, Seattle, WA, USA 3. Department of Cell and Tissue Biology, University of California San Francisco, San Francisco, CA, USA 4. Marine Biological Laboratory, Woods Hole, MA 02543, USA &. Current address: Harvard Medical School, Department of Systems Biology, Boston MA 02115 *. Authors had equal contributions to the work. #. Authors had equal contributions to the work.
Corresponding authors: rikkimgarner@gmail.com (R.M.G.), a.t.molines@gmail.com (A.T.M.)

Supplementary Figures

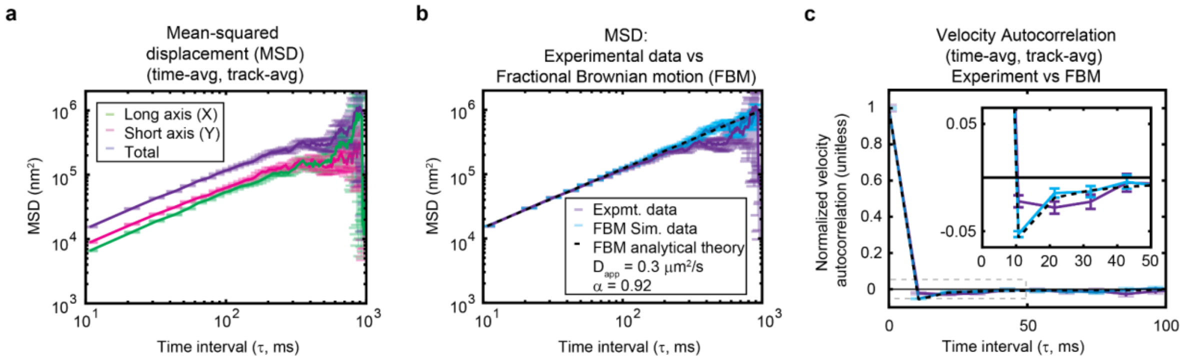

Supp. Fig. 1: Experimental data is consistent with nearly unconstrained diffusion. (**a**) Mean-squared displacement (MSD) along the long and short axes of the cell, plotted alongside the total MSD. (**b**) The predicted MSD for Fractional Brownian motion (FBM), including both analytical theory and results from simulated data, using the experimentally-measured values of D and α. The experimental data is also plotted for comparison, showing good agreement with the theory. (**a-b**) Note the logarithmic scale along the x- and y-axes. (**c**) Same legend as in (b). The predicted velocity autocorrelation for FBM, showing the characteristic negative peak which then decays to zero. Experimental data shows a wide and very shallow negative basin, which does not match the shape or depth of the peak predicted by FBM.

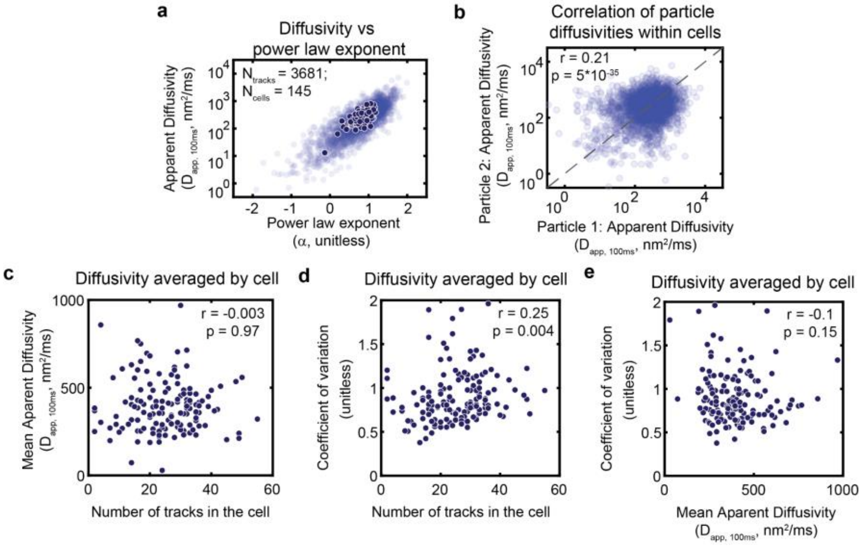

Supp. Fig. 2: Additional evidence for intrinsic and extrinsic sources of noise. (**a**) The relationship between the apparent diffusivity and power law exponent. (**b**) The apparent diffusivity of each particle in the dataset plotted against a randomly chosen particle from the same cell. Each particle is represented exactly once in the plot. For cells with an odd number of particles, one particle would not be represented for that cell. (**c**) Mean diffusivity across tracks in each cell plotted vs the number of tracks in each cell. (**d**) Coefficient of variation across tracks in each cell plotted vs the number of tracks in each cell. (**e**) Coefficient of variation vs mean diffusivity calculated by averaging across all tracks for each cell. (**a-e**) Fits of track-wise MSD data are shown in light blue, with cell-wise fits overlaid in dark blue. (**a-b**) Note the logarithmic scale along the y-axis. (**b**) Note the logarithmic scale along the x-axis. (**b-d**) r- and p-values determined by a Spearman correlation algorithm.

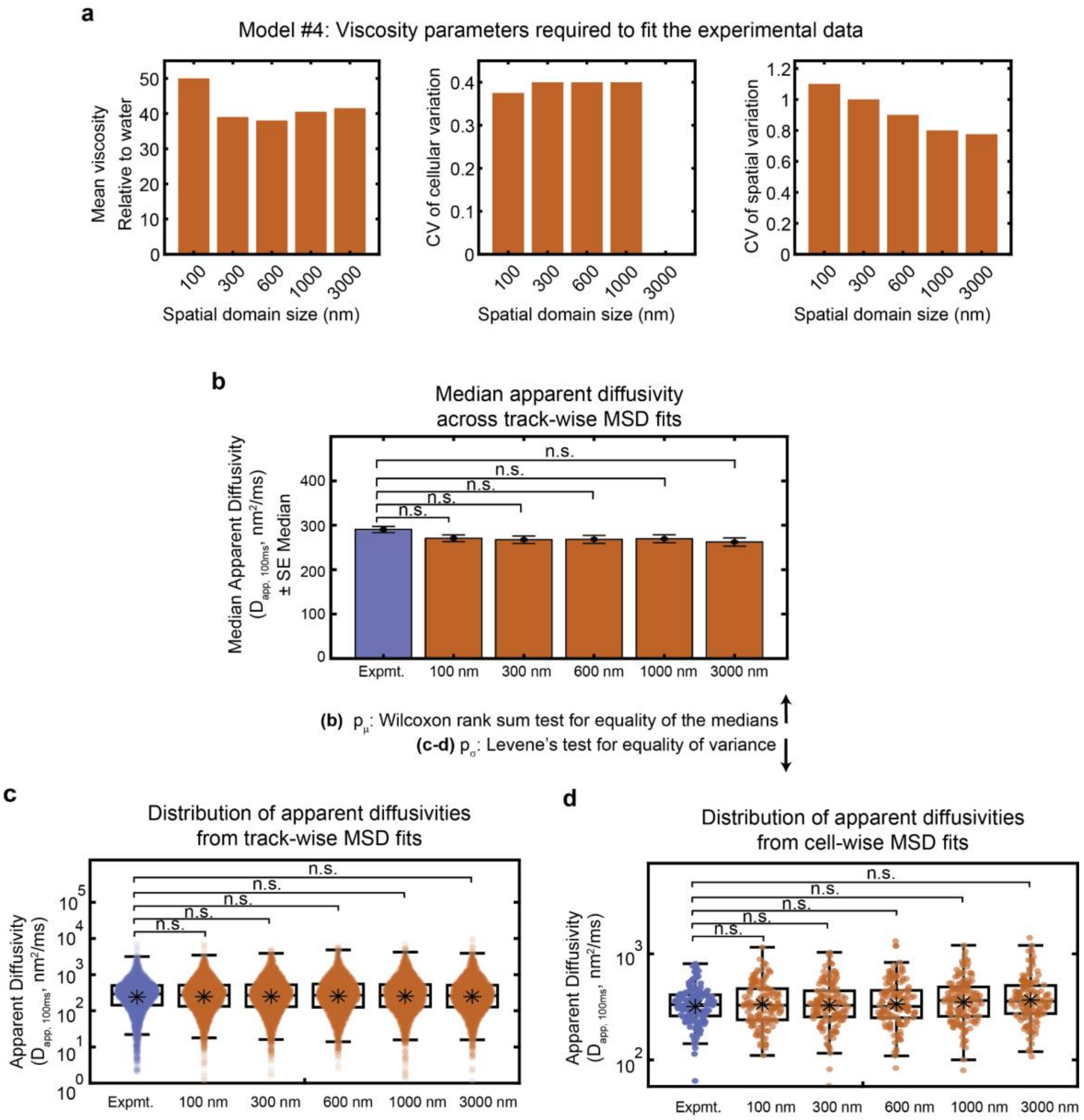

Supp. Fig. 3: Best fit parameters for each spatial domain size preserve the experimentally-observed mean and variance in diffusivity. (**a**) Simulation input parameters for viscosity (Model #4: Spatial and cellular heterogeneity) that best recapitulate the experimentally-measured spread in diffusivity. *Left*: The mean viscosity relative to the viscosity of water (e.g., A mean of 40 would indicate the cytoplasm has 40X the viscosity of water). *Middle*: The coefficient of variation (CV, mean divided by the standard deviation) of the viscosity among different spatial domains within each cell. *Right*: The coefficient of variation (CV, mean divided by the standard deviation) of the cell-averaged viscosities among a population of cells. (**b**) Median apparent diffusivity (averaged across all tracks) plotted for the experimental dataset as well as each model. X-labels for the models represent the domain size for the spatial heterogeneity. Error bars represent the standard error of the median. Significance stars represent the result of the Wilcoxon rank sum test for equality of the medians. (**c**-**d**) Distributions of apparent diffusivities calculated from fits of the track-wise (**c**) or cell-wise (**d**) MSD curves displayed for the experimental data as well as each of the models. Note the logarithmic scale along the y-axis. Boxplots are drawn as in Figure 2. Significance stars represent the result of Levene's test for equality of variance. (**a**-**c**) * $p<0.05$. ** $p<0.01$, *** $p<0.001$, **** $p<0.0001$.

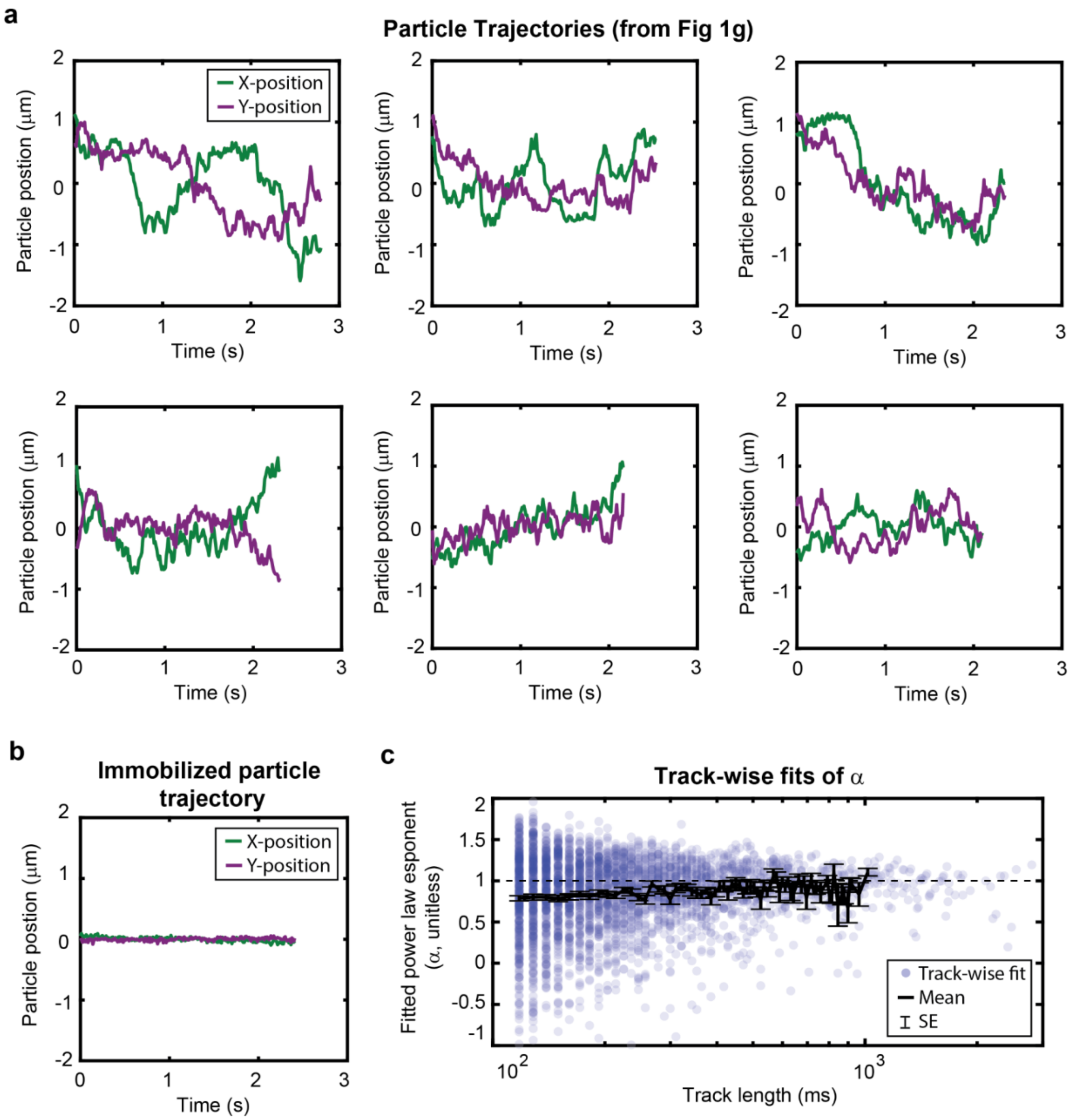

Supp. Fig. 4: Weak non-ergodicity of GEM diffusion cannot be explained by a continuous time random walk model. (**a**) X- and y-trajectories of the tracks shown in Fig. 1g. (**b**) X- and y-trajectories of a completely immobilized particle observed within the experimental dataset. (**c**) The best fit of the power law exponent, α, for time-averaged MSD of each track, plotted as a function of the track length. Each dot represents the best fit for an individual track. The mean across all tracks of a given length is displayed as a thick black line, and the standard error of the mean (SE) is plotted as error bars.

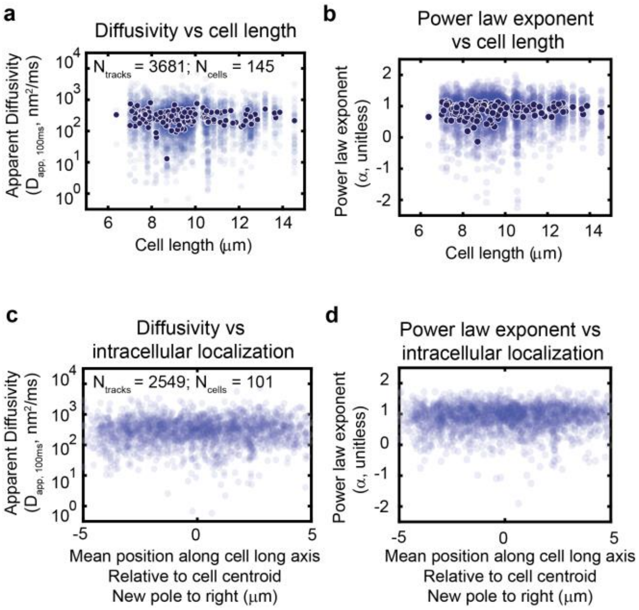

Supp. Fig. 5: The large heterogeneity in diffusivity cannot be explained by the cell cycle or subcellular GEM particle localization. (**a**-**b**) Fitted values for diffusivity (a) and power law exponent (**b**) plotted as a function of cell length. (**c**-**d**) Track-wise fit values for diffusivity (**c**) and power law exponent (**d**) plotted against the mean (time-averaged) particle position along the long axis of the cell. There are fewer cells and tracks represented in (**c-e**) compared to (**a-b**) because the new pole could be distinguished from the old pole for only a subset of cells. (**a-d**) Fits of track-wise MSD data are shown in light blue, with cell-wise fits overlaid in dark blue. (**a, c**) Note the logarithmic scale along the y-axis.

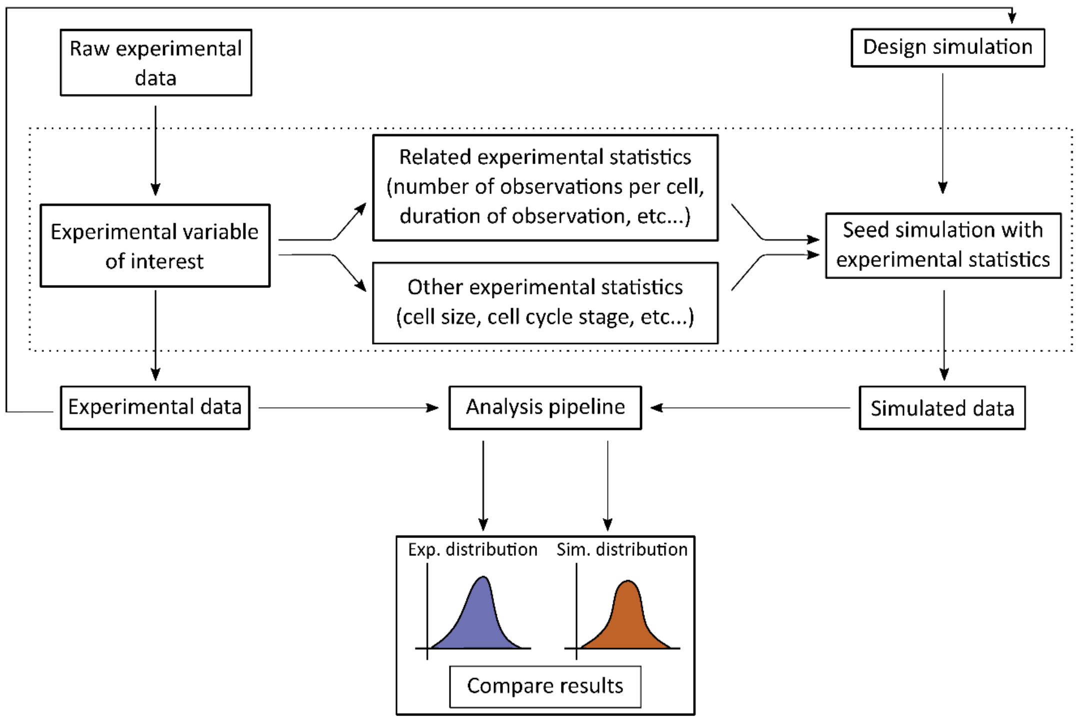

Supp. Fig. 6: Schematic of the generalized Doppelgänger approach.
